# Fermi Level Tuning of ZnO Films Through Supercycled Atomic Layer Deposition

**DOI:** 10.1186/s11671-017-2308-1

**Published:** 2017-09-19

**Authors:** Ruomeng Huang, Sheng Ye, Kai Sun, Kian S. Kiang, C. H. (Kees) de Groot

**Affiliations:** 1 Nanoelectronics and Nanotechnology Group, Department of Electronics and Computer Science, University of Southampton, Southampton, SO17 1BJ UK; 2 Southampton Nanofabrication Centre, University of Southampton, Southampton, SO17 1BJ UK

**Keywords:** Fermi level, ZnO, Atomic layer deposition, Kelvin probe force microscopy

## Abstract

A novel supercycled atomic layer deposition (ALD) process which combines thermal ALD process with in situ O_2_ plasma treatment is presented in this work to deposit ZnO thin films with highly tunable electrical properties. Both O_2_ plasma time and the number of thermal ALD cycles in a supercycle can be adjusted to achieve fine tuning of film resistivity and carrier concentration up to six orders of magnitude without extrinsic doping. The concentration of hydrogen defects are believed to play a major role in adjusting the electrical properties of ZnO films. Kelvin probe force microscopy results evidently show the shift of Fermi level in different ZnO films and are well associated with the changing of carrier concentration. This reliable and robust technique reported here clearly points towards the capability of using this method to produce ZnO films with controlled properties in different applications.

**Electronic supplementary material:**

The online version of this article (10.1186/s11671-017-2308-1) contains supplementary material, which is available to authorized users.

## Background

Once defined as the *future material*, zinc oxide (ZnO) has attracted the interest of science community for over half a century due to its superior optical and electrical properties [1]. Recently, the rapid growth of transparent conductive oxide industry has further revived its application as transparent electrodes in flat panel displays, touch screens, low emissivity coating, thin film solar cells, etc. [2, 3]. Furthermore, ZnO has found numerous applications in electronic devices including light-emitting diodes, photo detectors, and power devices [4, 5]. These different types of applications require ZnO films to have various electrical parameters, and some applications even demand multi-layer of ZnO films with different electrical properties [6]. For example, numerous efforts have been made to develop transparent resistive random access memory (TRRAM) for the realization of fully integrated transparent electronics [7, 8]. As one of the most promising candidates, ZnO-based TRRAM uses a highly resistive ZnO film as the active switching layer while highly conductive ZnO films are ideally demanded to act as transparent electrodes [8–10]. The capability of controlling the electrical properties such as resistivity and carrier concentration of the ZnO films is therefore a key requirement. Doping is usually used when the property modification is needed and a variety of dopants have been applied to change ZnO film properties [11–13]. However, doping is always complex and could lead to the secondary phase formation [14]. Modulation of the electrical properties of undoped ZnO by a single deposition process can therefore be advantageous.

Atomic layer deposition (ALD) has become a popular technique to form high-quality ZnO with an excellent control of the film thickness down to nanometer scale and uniformity over a large substrate [15, 16]. The growth temperature of ZnO is usually under 200 °C which makes it compatible with a range of substrates including glass and plastics. The ALD ZnO is normally grown by using diethylzinc (DEZ) as a Zn precursor and water vapor (thermal) or oxygen plasma (plasma-enhanced) as oxygen precursor. The dominant way of tuning undoped ZnO film properties in the thermal ALD process is by changing the growth temperature [17, 18]. Although this enables the deposition of highly conductive films, high-quality ZnO films are difficult to obtain with low carrier concentration. The plasma-enhanced ALD is preferably used when low-carrier concentration ZnO is required [19, 20]. We recently reported the capability of tuning ZnO using a single plasma-enhanced ALD process which allows the tuning of its resistivity and carrier concentration up to three orders by using different O_2_ plasma times [21]. However, the plasma-enhanced ALD could suffer a non-self-limiting growth if a short O_2_ plasma time is applied to achieve needed carrier concentration, which can result in poor uniformity over a large substrate. A tunable ALD process within the self-limiting window would therefore be desired.

Apart from the capability in tuning the ZnO electrical properties, determination of these properties also remains challenging. Hall effect measurement is the most popular technique in measuring the electrical properties of ZnO thin films. However, it can be prone to misinterpretation and has difficulty in unambiguous detection of the actual cause of doping [1]. Kelvin probe force microscopy (KPFM) is a non-destructive surface technique which has been extensively used to characterize nanoscale structural, dynamic, and electrical properties of semiconductor materials and devices [22, 23]. By directly measuring the contact potential difference (*V*_CPD_), i.e., the difference between the work functions of the tip and the sample, it can provide an insight into the material dopant types, carrier concentrations, and resistivity as they affect the Fermi level position within the bandgap. However, works correlating the ZnO properties with KPFM results are rarely reported, and to our knowledge, there are none based on ALD-grown ZnO films [24–26].

In this work, we propose a novel supercycled ALD process for electrical properties tuning in undoped ZnO. Combining the thermal ALD process with an in situ O_2_ plasma treatment, this process allows a wide yet refined tuning of the ZnO film resistivity and carrier concentration. More importantly, the Fermi level shifts in the ZnO films can be directly measured by KPFM and used to characterize the ZnO electrical properties.

## Methods

**Fig. 1:**
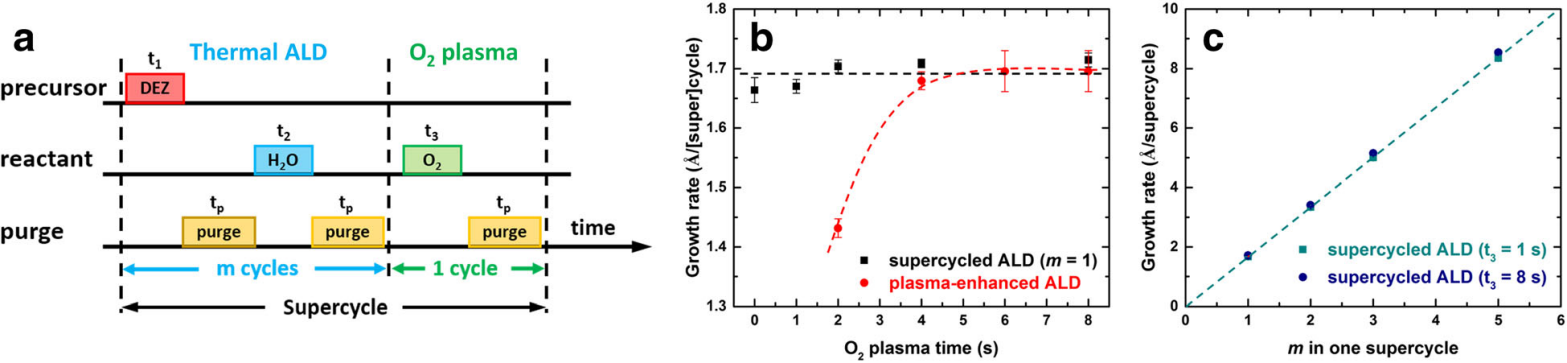
**a** Illustration of the one growth supercycle of the proposed supercycled ALD process. **b** ZnO growth rates as a function of the O_2_ plasma time for supercycled ALD with fixed thermal cycle (*m* = 1) and plasma-enhanced ALD processes; the dashed curves are guides of the eye. **c** ZnO growth rate and linear fitting as a function of the thermal process cycle *m* with fixed O_2_ plasma times (*t*_3_ = 1 and 8 s)

All ZnO thin films were prepared in an OIPT FlexAl ALD system using a diethylzinc (DEZ) precursor. Each supercycle of the ALD process consists of *m* cycles of thermal ALD processes (DEZ and H_2_O) and one O_2_ plasma step as demonstrated in Fig. 1a. Within the thermal ALD process, DEZ vapor was initially introduced into the chamber and then purged by an argon flow, and H_2_O vapor was subsequently introduced and then argon purged. After *m* cycles of thermal ALD processes, an O_2_ plasma step was added as an in situ plasma treatment step. The O_2_ plasma step was set using an O_2_ flow of 60 sccm, RF power of 300 W, and pressure of 15 mTorr. Both thermal ALD numbers (*m*) and O_2_ plasma time (*t*_3_) were used for ZnO film property control. The specific details for one growth supercycle in the ALD process are given in Additional file 1: Table S1. All ZnO films were deposited on the SiO_2_-coated Si substrates (1 cm × 1 cm) at a fixed temperature of 190 °C, and all film thicknesses were projected to be 40 nm.

The thickness and optical constants of the deposited ZnO films were measured by ellipsometry (VASE, J.A. Woollam Co. M-2000) and fitted with a Tauc-Lorentz (TL) model. The electrical properties were measured by Hall measurements (Nanometrics HL5500PC) at room temperature under a magnetic field of 0.5 T. Extra care was taken to ensure linear contact was obtained between each copper probe and the sample before every single measurement. X-ray diffraction (XRD) patterns were collected in grazing incidence (*θ*_1_ = 1°) using a Rigaku Smartlab diffractometer with a 9-kW Cu-*K*_α_ source. X-ray photoelectron spectroscopy (XPS) data were obtained using a Thermo Scientific Theta Probe System with Al-*K*_α_ radiation (photon energy = 1486.6 eV). Where necessary, surface contamination was eliminated by the use of an ion sputtering gun. The Zn 2*p*, O 1*s*, and C 1*s* spectra were collected. All data were referenced to the C 1*s* peak, which was assigned a binding energy of 284.6 eV. KPFM measurements were performed on Nanonics CV2000 by a Nanosensor ATEC Pt-Ir-coated tip with a resonant frequency of 65 kHz. To reduce the influence of surface contaminant, the measurements were carried out just after the samples were removed from the vacuum chamber.

## Results and Discussion

The proposed supercycled ALD process is illustrated in Fig. 1a with one supercycle consisting of *m* cycles of thermal ALD processes (DEZ and H_2_O) and one O_2_ plasma step (O_2_ plasma). More details are in the “Methods” section. Figure 1b compares the ZnO growth rates in our supercycled ALD process when *m* = 1 and the conventional plasma-enhanced ALD process as a function of O_2_ plasma time. The growth rate in the plasma-enhanced ALD process (red) is found to be sensitive to the O_2_ plasma as it increases from ca. 1.4 to 1.7 Å/cycle with the plasma time changing from 2 to 4 s. It is then saturated at the level of ca. 1.7 Å/cycle at longer plasma times. The unsaturated growth rate at shorter O_2_ plasma time is attributed to the oxygen deficiency in the process. Although this is sometimes preferred to obtain ZnO films with high conductivity, it is not self-limiting and could result in a poor uniformity over the whole substrate. On the other hand, the growth rate was found to be stable at ca. 1.69 Å/supercycle in the supercycled ALD process (black) and is close to that of the thermal ALD process (*t*_3_ = 0 s) regardless of the plasma time applied. Moreover, increase of the thermal cycle *m* in one supercycle with fixed plasma time leads to a linear increase of the growth rate as shown in Fig. 1c. The fitted gradient is calculated to be 1.67 for both growths with different O_2_ plasma times, which is also close to the growth rate of the thermal ALD process. This suggests the growth of ZnO in our supercycled ALD is dominated by the thermal ALD process and the subsequent O_2_ plasma step serves as merely a treatment.

**Fig. 2:**
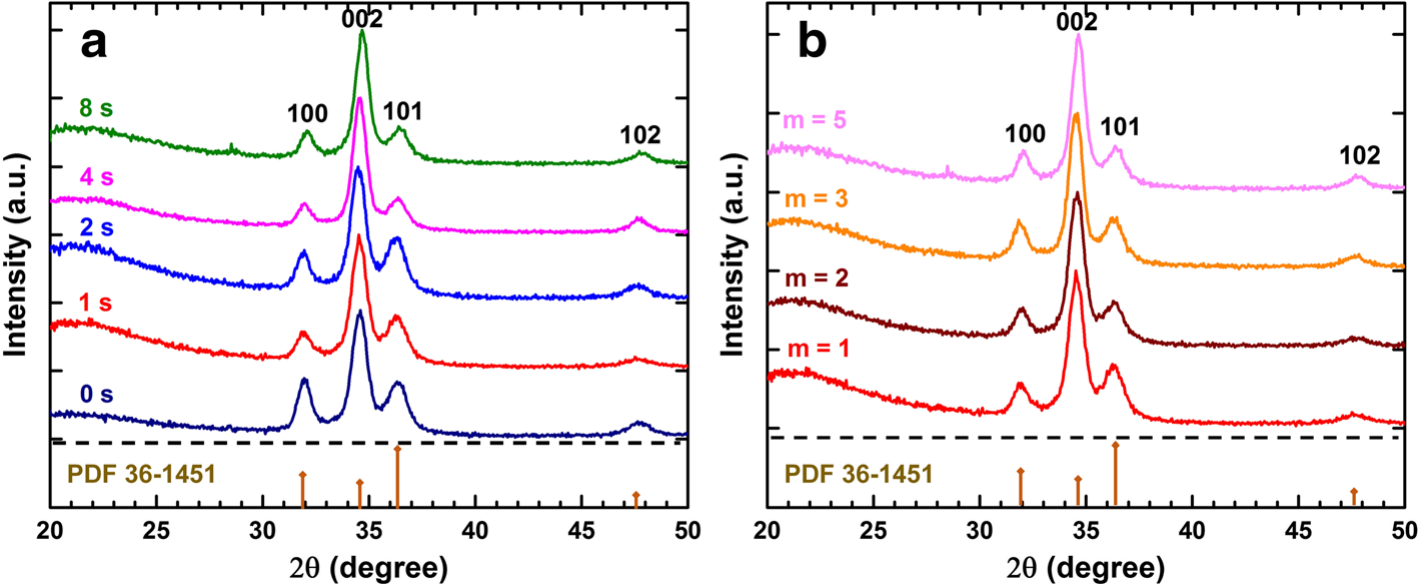
XRD patterns of ZnO films grown by the supercycled ALD process using **a** different O_2_ plasma times with fixed thermal cycle (*m* = 1) and **b** different thermal cycles with fixed O_2_ plasma time (*t*_3_ = 1 s)

All ZnO films grown by the supercycled ALD process (*m* = 1) crystallize in the hexagonal wurtzite structure and illustrate a similar distribution of peak intensities regardless of the O_2_ plasma time, as shown in Fig. 2a. Compared with the theoretical intensity ratio of 0.44 between peak (0 0 2) and (1 0 1) (calculated from JCPDS-34-1451 for a random orientation of the crystallites), these films demonstrate a strong preferred orientation along the *c*-axis with the (0 0 2) and (1 0 1) peak intensity ratio between 2 and 5, suggesting good crystalline quality of the films. A slight increase of the (0 0 2) to (1 0 1) peak ratio is observed with increasing O_2_ plasma time (shown in the Additional file 1: Figure S1). This suggests a higher degree of preferred orientation when exposed to longer plasma. Similar behavior was also reported [27, 28]. However, it is worth pointing out that the change of the intensity ratio in our work is rather trivial compared with others. This further suggests the stability of our supercycled ALD process for producing high quality ZnO films. The average grain sizes was also estimated based on the Scherrer formula [29] and were found to be ca. 11 nm, suggesting the ZnO grain size was barely affected by the O_2_ plasma time. Similar patterns are also observed on ZnO films grown from various thermal ALD cycles (*m*) with a fixed O_2_ plasma time (1 s) as shown in Fig. 2b.

Aside from the crystallinity, the optical properties of the supercycled ALD-grown ZnO films were also studied using spectroscopic ellipsometry (SE). Optical constants (*n* and *k*) can be extracted from the ellipsometry results by a Tauc-Lorentz model which is commonly used in fitting ZnO films [28, 30, 31]. Similar to the analogous crystallinity, the optical properties of ZnO films deposited with different O_2_ plasma times and thermal cycles also remain unchanged as shown in Additional file 1: Figure S2. This is consistent with the reported works that a change of crystallinity is always associated with a change of optical properties [28, 32]. The morphological properties of the ZnO films are characterized by AFM. All films were found to be similarly smooth with average roughness between ca. 0.3 and 0.8 nm (Additional file 1: Figure S3).

**Fig. 3:**
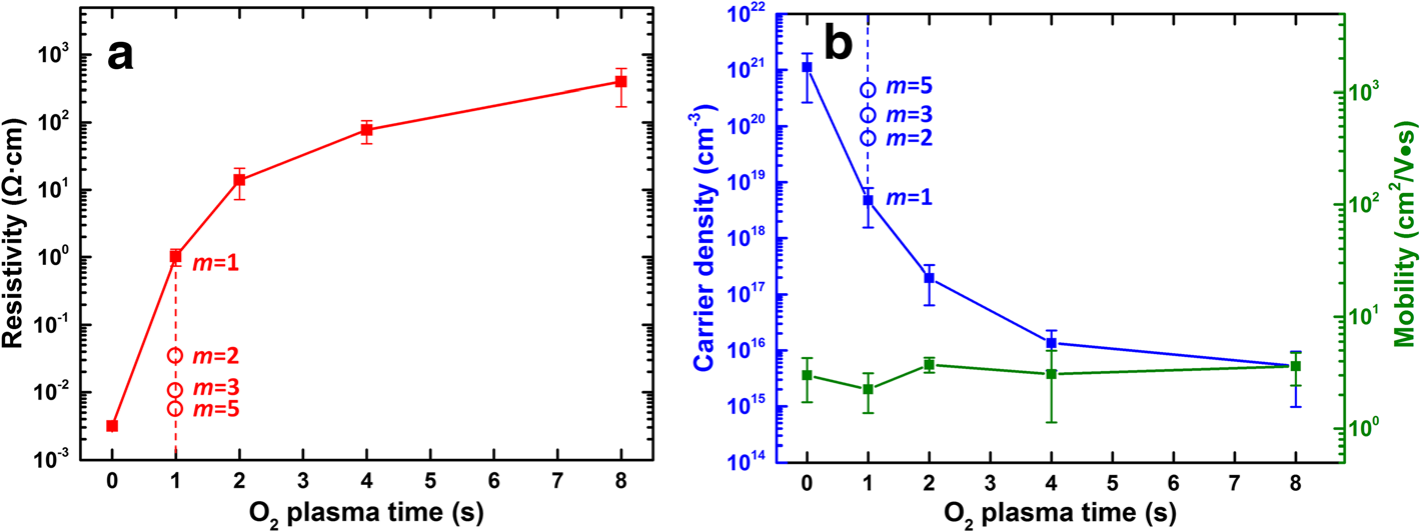
**a** Electrical resistivity of ZnO films grown from different O_2_ plasma times with fixed thermal cycle *m* (solid dots) and different thermal cycles with fixed O_2_ plasma time (open dots) by the supercycled ALD process. **b** Carrier concentration (blue) of ZnO films grown from different O_2_ plasma times with fixed thermal cycle (solid dots) and different thermal cycles with fixed O_2_ plasma time (open dots). Mobility (green) of ZnO films grown from different O_2_ plasma times with a fixed thermal cycle

The electrical properties of the ZnO films grown by the supercycled ALD process are investigated by a Hall effect system. All films were found to be *n*-type semiconducting, and the resistivity increases from ca. 10^−3^ to 10^3^ Ω cm with increasing O_2_ plasma time and fixed thermal cycle (*m* = 1) as shown in Fig. 3a. This is associated with the reduction of the carrier concentration from ca. 10^21^ to 10^15^ cm^−3^ as the O_2_ plasma time increases from 0 to 8 s (Fig. 3b). In contrary, the electron mobilities of all ZnO films are found to be rather consistent (ca. 3.0 ± 1.0 cm^2^/V s) and are not affected by the plasma duration. The detailed conducting mechanism will be addressed further in the section below. Compared with the plasma-enhanced ALD process we reported earlier [21], the magnitude of resistivity tuning has been further improved in the supercycled ALD process to over five orders. In addition, this proposed ALD process offers a more refined control over these electrical properties by varying the thermal cycle (*m*) in one supercycle while fixing the O_2_ plasma time (*t*_3_). This is particularly useful in the case of *t*_3_ = 1 s where tuning is not achievable by further reducing the plasma time due to the limitation of the ALD equipment. The open dots in Fig. 3a, b represent the resistivities and carrier concentrations of ZnO films grown by different thermal cycles (*m* = 2, 3, 5) when *t*_3_ = 1 s (error bars are within the dots). It can be observed that more thermal cycles result in less resistive films with higher carrier concentrations. This provides an extra of three resistivities within the range of 10^−3^ to 10^1^ Ω cm.

**Fig. 4:**
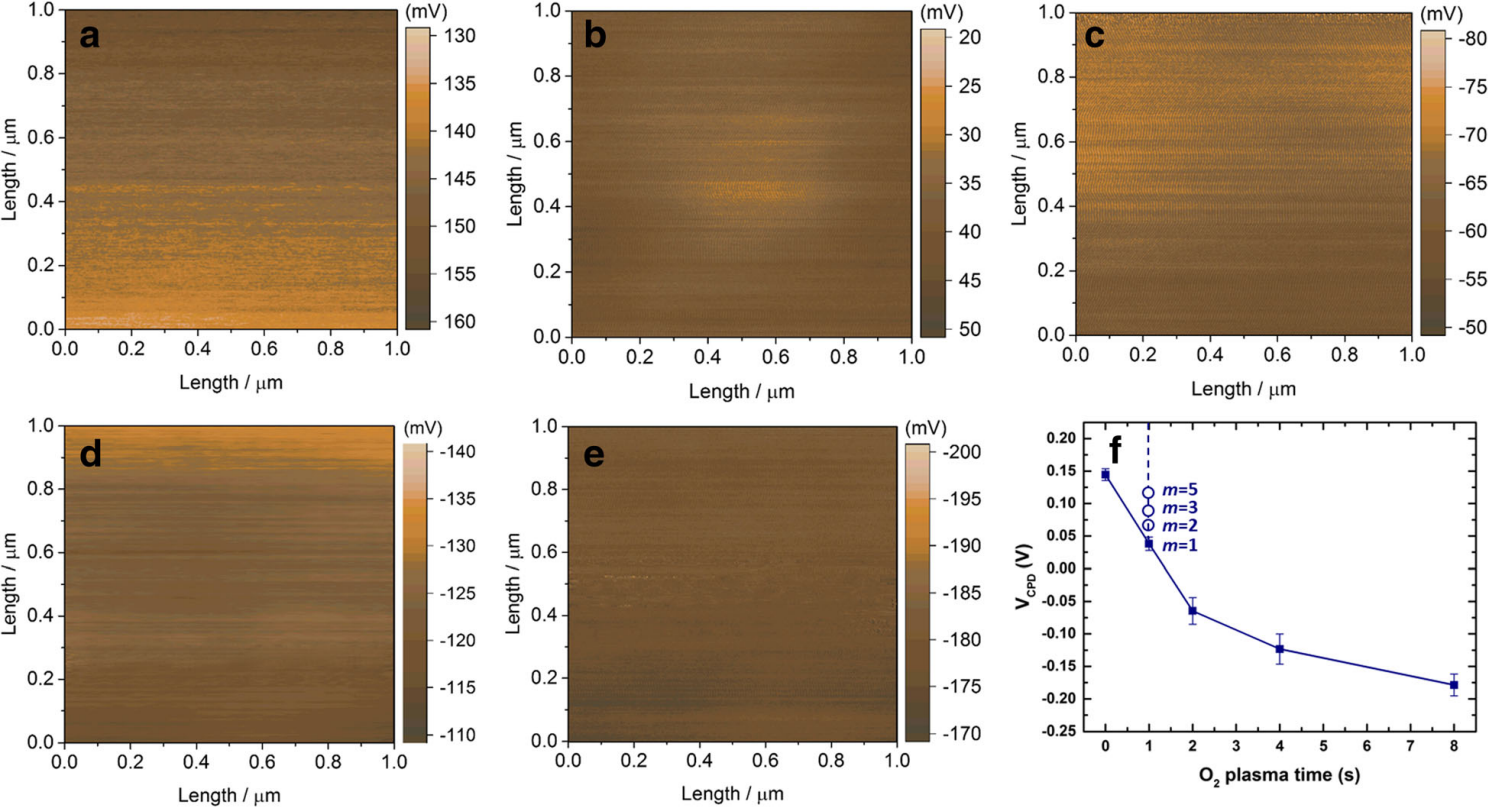
**a**–**e** Two-dimensional contact potential difference *V*_CPD_ images of the surface potential measurements of the supercycled ALD-grown ZnO films with O_2_ plasma time (*t*_3_) varying from 0 to 8 s and fixed thermal cycle (*m* = 1). **f** Average *V*_CPD_ values with varying O_2_ plasma times (solid dots) and thermal cycles (open dots)

KPFM measurements were performed to gain insights into the Fermi level positions of the ZnO films with changing resistivity. It measures the contact potential difference *V*_CPD_ between a conductive tip and the sample which is defined as:

1 $$ {V}_{\mathrm{CPD}}=\frac{\phi_{\mathrm{tip}}-{\phi}_{\mathrm{sample}}}{q} $$

where *q* is the electronic charge, while *ϕ*_tip_ and *ϕ*_sample_ are the work function of the tip and the sample respectively. When two different materials are brought into electric contact, the Fermi levels will line up through electron current flow which consequently induces a contact potential difference between the tip and sample as shown in Additional file 1: Figure S4. Detailed working principle of KPFM is presented in Additional file 1. The contact potential differences of the ZnO films grown by the supercycled ALD process from different O_2_ plasma times with fixed thermal cycle (*m* = 1) are shown in Fig. 4. While each *V*_CPD_ image appears to be uniform and relatively smooth, substantial differences in the mean *V*_CPD_ values can be observed (shown in Fig. 4f). The tip work function *ϕ*_tip_ remains constant for all measurements; the substantial difference in *V*_CPD_ is therefore the consequence of the Fermi level shift within different ZnO films. A total shift of ca. 0.32 eV is obtained between ZnO film grown with 0 and 8 s O_2_ plasma time which is significant compared to the ZnO bandgap (ca. 3.22 eV based on the SE results in this work as shown in Additional file 1: Figure S2c). For ZnO films grown from different thermal cycles (*m* = 2, 3, 5) at fixed O_2_ plasma time (*t*_3_ = 1 s), different *V*_CPD_ values were also detected as shown in Fig. 4f. The two-dimensional KPFM images of these films can be found in Additional file 1: Figure S5. This implies the change in the electron-hole balance occurs throughout the films which could make considerable impact to the ZnO carrier concentration.

**Fig. 5:**
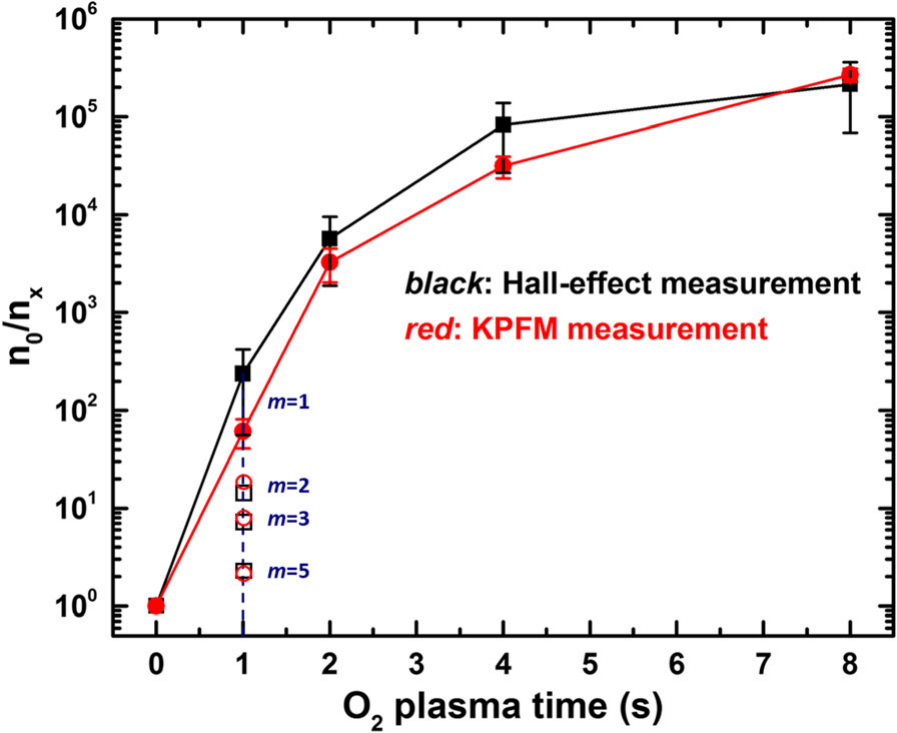
Hall effect and KPFM measurement results of carrier concentration ratios between ZnO films grown with varying O_2_ plasma times (solid dots) and thermal cycles (open dots)

In order to investigate the relation between the Fermi level and carrier concentration, we adopt the electronic energy model proposed by Maragliano et al. to correlate the contact potential difference *V*_CPD_ with the doping concentration in the material [26]. Assuming the effective donor concentration *n*_D_ is significantly higher than the intrinsic carrier concentration, it can be written as:

2 $$ {n}_{\mathrm{D}}\approx {N}_{\mathrm{C}}\exp \left(\frac{q{V}_{\mathrm{C}\mathrm{PD}}-{\phi}_{\mathrm{tip}}+\chi }{K_BT}\right) $$

where *N*_C_ is the effective density of states, *χ* is the electron affinity of the semiconductor, *K*_B_ is the Boltzmann constant, and *T* is the temperature. Although the values of the effective density of states *N*_C_, the tip work function *ϕ*_tip_, and the electron affinity *χ* are difficult to obtain, the relative carrier concentration differences of different ZnO films can be calculated as these values are the same in all measurements. Hence, the carrier concentration ratio between the films grown with 0 s of O_2_ plasma time to a given ZnO film can be expressed as:

3 $$ \frac{n_0}{n_x}=\exp \left(\frac{V_{\mathrm{CPD}0}-{V}_{\mathrm{CPD}x}}{K_BT/q}\right) $$

in which *n*_0_ and *n*_*x*_ are the carrier concentration of the ZnO film grown with 0 and *x* s of O_2_ plasma time, respectively, and *V*_CPD0_ and *V*_CPD*x*_ are the corresponding contact potential differences. The calculated carrier concentration ratios are plotted in Fig. 5 as a function of O_2_ plasma time. The concentration ratio is calculated to increase with longer O_2_ plasma time (red). More importantly, the increasing trend matches well with the values obtained from the Hall effect measurement results (black). Similar trend was also observed for ZnO films grown from different thermal cycles (*m* = 2, 3, 5) at fixed O_2_ plasma time (*t*_3_ = 1 s). This evidently suggests the shift of ZnO film Fermi level is directly associated with the carrier concentration level.

**Fig. 6:**
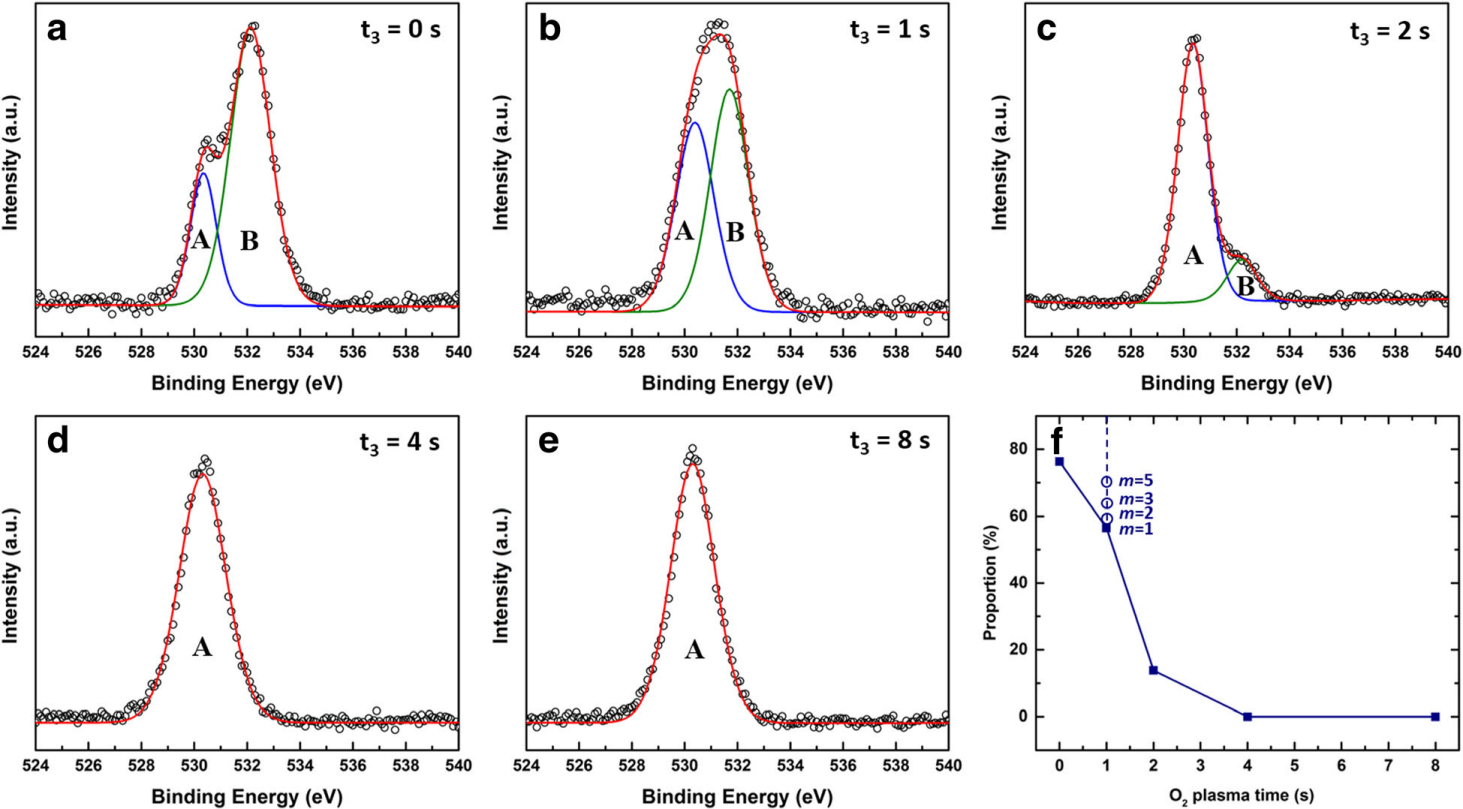
**a**–**e** XPS spectra and their Gaussian fittings of the O 1*s* region of the supercycled ALD-grown ZnO films with O_2_ plasma time (*t*_3_) varying from 0 to 8 s and fixed thermal cycle (*m* = 1). **f** Proportion of the hydrogen-related O_B_ peak in ZnO films grown with varying O_2_ plasma times (solid dots) and thermal cycles (open dots)

X-ray photoelectron spectroscopy (XPS) measurements were carried out to shed light on the conduction mechanism by studying the bonding and chemical states of the supercycled ALD-grown ZnO films. The chemical states of O 1*s* are shown in Fig. 6 where two peaks can be identified after Gaussian fittings. The lower energy peak (A) positioned ca. 530.3 eV is suggested to be the O^2−^ ions in the wurtzite structure of hexagonal Zn^2+^ ions [33–35]. The assignments of the higher bonding energy component at ca. 532.2 (B) have been controversial over literatures [33–38]. However, it is widely reported that it is associated with the hydroxyl group (i.e., Zn–OH) [33, 37–39] in ZnO film. We therefore tentatively ascribe the O_B_ peak observed in Fig. 6 to the Zn−OH bonds. On the other hand, the oxygen vacancy-associated peak positioned at ca. 531.2 eV [35] is not observed in this work. ZnO film grown by the thermal ALD process (*t*_3_ = 0 s) characterizes a dominant O_B_ peak in the XPS spectrum (Fig. 6a). This implies the existence of large amount of hydrogen-related impurities in this film. This high level of defects serves as a self-doping mechanism and leads to a high carrier concentration. The extra O_2_ plasma step (*t*_3_) diminishes the impurities, and the O_B_ peak intensity decreases with longer O_2_ plasma time (Fig. 6f). Similar trend was also observed for ZnO films grown from varying thermal cycles in which more thermal cycles lead to the increase of O_B_ peak intensity as shown in Fig. 6f and Additional file 1: Figure S6.

**Fig. 7:**
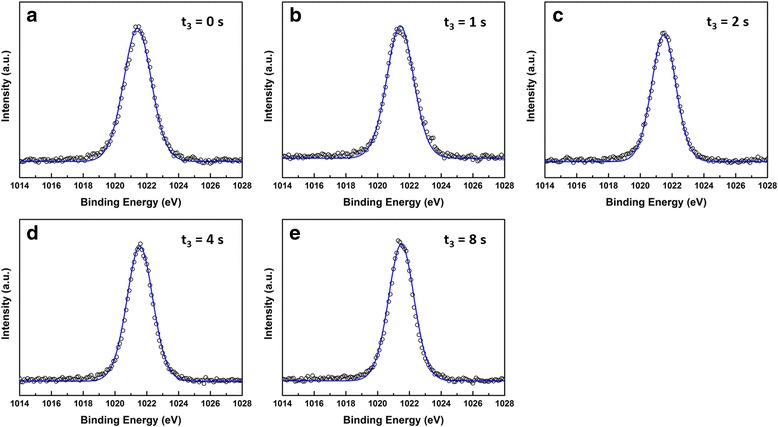
**a**–**e** XPS spectra and their Gaussian fittings of the Zn 2*p*_3/2_ region of the supercycled ALD-grown ZnO films with O_2_ plasma time (*t*_*3*_) varying from 0 to 8 s and fixed thermal cycle (*m* = 1)

The origin of the intrinsic *n*-type conductivity in ZnO films is still controversial. Although conventional wisdom has attributed this conductivity to native defect (i.e., oxygen vacancies and Zn interstitials) [18, 40–44], it has been challenged by recent first-principles calculations [45]. Oxygen vacancies are also unlikely to contribute to such a high concentration level as numerous studies suggest they are deep rather than shallow donors and have high formation energies in *n*-type ZnO (and are therefore unlike to form) [41–44]. Additionally, the oxygen vacancy-related O 1*s* peak is also not observed in our XPS data shown in Fig. 6. Although Zn interstitials are shallow donors, they have been suggested to have high formation energies and are faster diffusers and hence are unlikely to be stable [41]. The XPS spectra of the Zn 2*p*_3/2_ state of the supercycled ALD-grown ZnO films with various O_2_ plasma times are shown Fig. 7. All spectra are characterized by a similar peak positioned at ca. 1021.5 eV which can be attributed to the Zn^2+^ bonding in ZnO [6, 34, 46]. However, the Zn interstitial component at a slightly higher binding energy [6, 47] is not observed in all spectra. This suggests that the influence of Zn interstitial on the ZnO film conductivity can also be ruled out in this work.

**Fig. 8:**
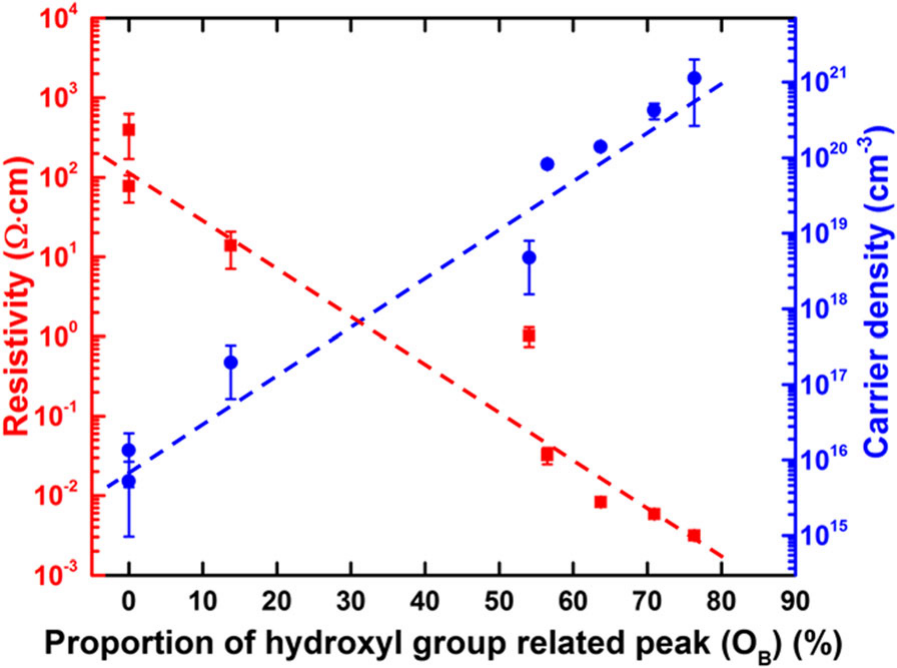
Electrical resistivity and carrier concentration as a function of the hydroxyl group-related O_B_ peak proportion (dashed lines are guides of the eye)

Recently, hydrogen-related impurities/defects are proposed to play a role in the *n*-type conductivity in ZnO [33, 48]. The evidence of the existence of hydrogen bonds in ZnO has been demonstrated by Janotti et al. [48], and it have been suggested that those bonds are able to incorporate in high concentrations and behave as shallow donors [49–51]. Indeed, hydrogen is present in our supercycled ALD process as both precursor and H_2_O contain hydrogen and a Zn−OH bond is produced in every half cycle in the thermal ALD step. This is also supported by the observation of hydroxyl group-related O 1*s* peak in the XPS spectra (shown in Fig. 6). The ZnO film resistivity and carrier density are plotted against the proportion of this peak in Fig. 8. High proportion of these hydrogen impurities induce high carrier concentrations, leading to low resistivities. The subsequent O_2_ plasma step within each supercycle reduces the carrier concentration by effectively removing the hydrogen bonds. This is accompanied by the reduction of carrier concentration as well as the increase of resistivity. Same behavior was also reported on both ALD and CVD processes [33, 52].

## Conclusions

Deposition of undoped ZnO films with highly tunable electrical properties was reported here using a supercycled ALD process which combines a thermal ALD process and in situ O_2_ plasma treatment. More than five orders of magnitude adjustment over the film resistivity and carrier concentration were achieved by tuning the O_2_ plasma times. Finer tuning of the properties can also be realized by varying the number of thermal ALD cycles in a supercycle. The tuning of these electrical properties is believed to be associated with the change of hydrogen defect concentration in the film. This directly causes the shift of Fermi levels within the ZnO films as revealed by the Kelvin probe force microscopy. By adopting a simple electronic energy model, the carrier concentrations calculated from the Fermi level shifts demonstrate a good match with the Hall effect measurement results. This reliable and robust technique reported here clearly points towards the capability of using this method to produce ZnO films with controlled properties in different applications.

## Additional files

Additional file 1: Table S1. Process details for one growth supercycle in the supercycled ALD process used in this study. **Figure S1.** Diffraction intensity ratio of the ZnO (0 0 2) peak to (1 0 1) peak as a function of O_2_ plasma times with fixed thermal cycle (*m* = 1). **Figure S2.** (a, d) The fitted refractive index *n*, (b, e) extinction coefficient *k*, and (c, f) plot of (*αhν*)^2^ as a function of photo energy of ZnO films grown from different O_2_ plasma times with fixed thermal cycle (*m* = 1) and different thermal cycles with fixed O_2_ plasma time (*t*_3_ = 1 s) by the supercycled ALD process. **Figure S3.** AFM images of the supercycled ALD-grown ZnO films with O_2_ plasma times of (a) 0 s, (b) 1 s, (c) 2 s, (d) 4 s, and (e) 8 s and fixed thermal cycle (*m* = 1). **Figure S4.** Electronic energy levels of the tip sample system for three different cases. (a) Tip and sample are not electrically connected; (b) tip and sample are electrically connected with Fermi energy levels lined up; (c) an external bias equals to the contact potential difference *V*_CPD_ is applied to the tip. **Figure S5.** Two-dimensional contact potential difference *V*_CPD_ images of the surface potential measurements of the supercycled ALD-grown ZnO films with thermal cycles varying from (a) 2, (b) 3, and (c) 5 at fixed O_2_ plasma time (*t*_3_ = 1 s). **Figure S6.** XPS spectra and their Gaussian fittings of the O 1*s* region of the supercycled ALD-grown ZnO films with thermal cycles varying from (a) 2, (b) 3, and (c) 5 at fixed O_2_ plasma time (*t*_3_ = 1 s). (DOCX 1919 kb)

## Abbreviations

ALD: Atomic layer deposition
DEZ: Diethylzinc
KPFM: Kelvin probe force microscopy
XPS: X-ray photoelectron spectroscopy
XRD: X-ray diffraction

## References

[1] Janotti A, Van de Walle CG (2009). Fundamentals of zinc oxide as a semiconductor. Reports Prog Phys.

[2] Edwards PP, Porch A, Jones MO et al (2004) Basic materials physics of transparent conducting oxides. Dalt Trans:2995–3002. doi:10.1039/b408864f 10.1039/b408864f 15452622

[3] Liu Y, Li Y, Zeng H (2013). ZnO-based transparent conductive thin films: doping, performance, and processing. J Nanomater.

[4] Ozgür M, Hofstetter D, Morkoç H (2010). ZnO devices and applications: a review of current status and future prospects. Proc IEEE.

[5] Fujita S (2015). Wide-bandgap semiconductor materials: for their full bloom. Jpn J Appl Phys.

[6] Guziewicz E, Godlewski M, Wachnicki L (2012). ALD grown zinc oxide with controllable electrical properties. Semicond Sci Technol.

[7] Thomas G (1997). Materials science: invisible circuits. Nature.

[8] Simanjuntak FM, Panda D, Wei K, Tseng T (2016). Status and prospects of ZnO-based resistive switching memory devices. Nanoscale Res Lett.

[9] Cao X, Li X, Gao X (2011). All-ZnO-based transparent resistance random access memory device fully fabricated at room temperature. J Phys D Appl Phys.

[10] Mundle R, Carvajal C, Pradhan AK (2016). ZnO/Al:ZnO transparent resistive switching devices grown by atomic layer deposition for memristor applications. Langmuir.

[11] Huang Q, Wang Y, Wang S (2012). Transparent conductive ZnO:B films deposited by magnetron sputtering. Thin Solid Films.

[12] Sans JA, Sánchez-Royo JF, Segura A (2009). Chemical effects on the optical band-gap of heavily doped ZnO:*M*_III_ (*M* = Al, Ga, In): an investigation by means of photoelectron spectroscopy, optical measurements under pressure, and band structure calculations. Phys Rev B.

[13] Bhachu DS, Sankar G, Parkin IP (2012). Aerosol assisted chemical vapor deposition of transparent conductive zinc oxide films. Chem Mater.

[14] Ellmer K, Bikowski A (2016). Intrinsic and extrinsic doping of ZnO and ZnO alloys. J Phys D Appl Phys.

[15] Johnson RW, Hultqvist A, Bent SF (2014). A brief review of atomic layer deposition: from fundamentals to applications. Mater Today.

[16] Tynell T, Karppinen M (2014). Atomic layer deposition of ZnO: a review. Semicond Sci Technol.

[17] Kowalik IA, Guziewicz E, Kopalko K (2009). Structural and optical properties of low-temperature ZnO films grown by atomic layer deposition with diethylzinc and water precursors. J Cryst Growth.

[18] Godlewski M, Guziewicz E, Szade J (2008). Vertically stacked non-volatile memory devices—material considerations. Microelectron Eng.

[19] Sun K, Zeimpekis I, Hu C (2016). Low-cost top-down zinc oxide nanowire sensors through a highly transferable ion beam etching for healthcare applications. Microelectron Eng.

[20] Sultan SM, Sun K, Clark OD (2012). Electrical characteristics of top-down ZnO nanowire transistors using remote plasma ALD. IEEE Electron Device Lett.

[21] Huang R, Sun K, Kiang KS (2016). Forming-free resistive switching of tunable ZnO films grown by atomic layer deposition. Microelectron Eng.

[22] Nonnenmacher M, O’Boyle MP, Wickramasinghe HK (1991). Kelvin probe force microscopy. Appl Phys Lett.

[23] Melitz W, Shen J, Kummel AC, Lee S (2011). Kelvin probe force microscopy and its application. Surf Sci Rep.

[24] Van Ben C, Cho HD, Kang TW, Yang W (2012). Doping transition of doped ZnO nanorods measured by Kelvin probe force microscopy. Thin Solid Films.

[25] Su T, Zhang H-F (2012). Influence of oxygen partial pressure on the Fermi level of ZnO films investigated by Kelvin probe force microscopy. Chinese Phys Lett.

[26] Maragliano C, Lilliu S, Dahlem MS (2014). Quantifying charge carrier concentration in ZnO thin films by scanning Kelvin probe microscopy. Sci Rep.

[27] Jin M, Jo J, Neupane GP (2013). Tuning of undoped ZnO thin film via plasma enhanced atomic layer deposition and its application for an inverted polymer solar cell. AIP Adv.

[28] Zhang JP, He G, Zhu LQ (2007). Effect of oxygen partial pressure on the structural and optical properties of ZnO film deposited by reactive sputtering. Appl Surf Sci.

[29] Patterson A (1939). The Scherrer formula for X-ray particle size determination. Phys Rev.

[30] Tan ST, Chen BJ, Sun XW (2005). Blueshift of optical band gap in ZnO thin films grown by metal-organic chemical-vapor deposition. J Appl Phys.

[31] Agocs E, Fodor B, Pollakowski B (2014). Approaches to calculate the dielectric function of ZnO around the band gap. Thin Solid Films.

[32] Cho EN, Park S, Yun I (2012). Spectroscopic ellipsometry modeling of ZnO thin films with various O_2_ partial pressures. Curr Appl Phys.

[33] Thomas MA, Cui JB (2012). Highly tunable electrical properties in undoped ZnO grown by plasma enhanced thermal-atomic layer deposition. ACS Appl Mater Interfaces.

[34] Ansari SA, Khan MM, Kalathil S (2013). Oxygen vacancy induced band gap narrowing of ZnO nanostructures by an electrochemically active biofilm. Nano.

[35] Zhang X, Qin J, Xue Y (2014). Effect of aspect ratio and surface defects on the photocatalytic activity of ZnO nanorods. Sci Rep.

[36] Ahn CH, Kim JH, Cho HK (2012). Tunable electrical and optical properties in composition controlled Hf:ZnO thin films grown by atomic layer deposition. J Electrochem Soc.

[37] Chen M, Wang X, Yu Y (2000). X-ray photoelectron spectroscopy and auger electron spectroscopy studies of Al-doped ZnO films. Appl Surf Sci.

[38] Hsu J-C, Lin Y-H, Wang PW, Chen Y-Y (2012). Spectroscopic ellipsometry studies on various zinc oxide films deposited by ion beam sputtering at room temperature. Appl Opt.

[39] Park S-M, Ikegami T, Ebihara K (2006). Effects of substrate temperature on the properties of Ga-doped ZnO by pulsed laser deposition. Thin Solid Films.

[40] Chang C-Y, Tsai F-Y (2011). Efficient and air-stable plastics-based polymer solar cells enabled by atomic layer deposition. J Mater Chem.

[41] Janotti A, Van de Walle CG (2007). Native point defects in ZnO. Phys Rev B.

[42] Look DC, Hemsky JW, Sizelove JR (1999). Residual native shallow donor in ZnO. Phys Rev Lett.

[43] Zhang SB, Wei S-H, Zunger A (2001). Intrinsic n-type versus p-type doping asymmetry and the defect physics of ZnO. Phys Rev B.

[44] Kohan A, Ceder G, Morgan D, Van de Walle C (2000). First-principles study of native point defects in ZnO. Phys Rev B.

[45] Janotti A, Van de Walle CG (2005). Oxygen vacancies in ZnO. Appl Phys Lett.

[46] Lupan O, Pauporté T, Chow L (2010). Effects of annealing on properties of ZnO thin films prepared by electrochemical deposition in chloride medium. Appl Surf Sci.

[47] Kayaci F, Vempati S, Donmez I (2014). Role of zinc interstitials and oxygen vacancies of ZnO in photocatalysis: a bottom-up approach to control defect density. Nano.

[48] Janotti A, Van de Walle CG (2007). Hydrogen multicentre bonds. Nat Mater.

[49] Van De Walle CG (2000). Hydrogen as a cause of doping in zinc oxide. Phys Rev Lett.

[50] Wardle MG, Goss JP, Briddon PR (2005). Theory of Fe, Co, Ni, Cu, and their complexes with hydrogen in ZnO. Phys Rev B.

[51] Lavrov E, Börrnert F, Weber J (2005). Photoconductivity and infrared absorption study of hydrogen-related shallow donors in ZnO. Phys Rev B.

[52] Hlaing Oo WM, McCluskey MD, Huso J, Bergman L (2007). Infrared and Raman spectroscopy of ZnO nanoparticles annealed in hydrogen. J Appl Phys.

