# Supplementary material for: Fermi Level Tuning of ZnO Films Through Supercycled Atomic Layer Deposition
Source: Nanoscale Res Lett. 2017 Sep 19;12:541. doi: 10.1186/s11671-017-2308-1 (PMC5605484; doi:10.1186/s11671-017-2308-1)
Supplement: Supplementary file 1 — Process details for one growth supercycle in the supercycled ALD process used in this study. Figure S1. Diffraction intensity ratio of the ZnO (0 0 2) peak to (1 0 1) peak as a function of O2 plasma times with fixed thermal cycle (m = 1). Figure S2. (a, d) The fitted refractive index n, (b, e) extinction coefficient k, and (c, f) plot of (αhν)2 as a function of photo energy of ZnO films grown from different O2 plasma times with fixed thermal cycle (m = 1) and different thermal cycles with fixed O2 plasma time (t 3 = 1 s) by the supercycled ALD process. Figure S3. AFM images of the supercycled ALD-grown ZnO films with O2 plasma times of (a) 0 s, (b) 1 s, (c) 2 s, (d) 4 s, and (e) 8 s and fixed thermal cycle (m = 1). Figure S4. Electronic energy levels of the tip sample system for three different cases. (a) Tip and sample are not electrically connected; (b) tip and sample are electrically connected with Fermi energy levels lined up; (c) an external bias equals to the contact potential difference V CPD is applied to the tip. Figure S5. Two-dimensional contact potential difference V CPD images of the surface potential measurements of the supercycled ALD-grown ZnO films with thermal cycles varying from (a) 2, (b) 3, and (c) 5 at fixed O2 plasma time (t 3 = 1 s). Figure S6. XPS spectra and their Gaussian fittings of the O 1s region of the supercycled ALD-grown ZnO films with thermal cycles varying from (a) 2, (b) 3, and (c) 5 at fixed O2 plasma time (t 3 = 1 s). (DOCX 1919 kb) [file 11671_2017_2308_MOESM1_ESM.docx]

**Fermi level tuning of ZnO films through supercycled atomic layer deposition**

**Ruomeng Huang^1^, Sheng Ye^1^, Kai Sun^1^, Kian S. Kiang^2^ and C. H. (Kees) de Groot^1^**

^1^ Electronics and Computer Science, University of Southampton, SO17 1BJ UK.

^2^ Southampton Nanofabrication Centre, University of Southampton, SO17 1BJ UK

| **Parameter** | **Condition** |
| --- | --- |
| DEZ pulse **t_1_** | 50 (ms) |
| H_2_O pulse **t_2_** | 150 (ms) |
| O_2_ plasma time **t_3_** | 0, 1, 2, 4, 8 (s) |
| Purge time **t_p_** | 5 (s) |
| Thermal-ALD cycle ***m*** | 1, 2, 3, 5 |
| O_2_ plasma power **P** | 300 (W) |

**Table 1.** Process details for one growth supercycle in the supercycled ALD process used in this study.


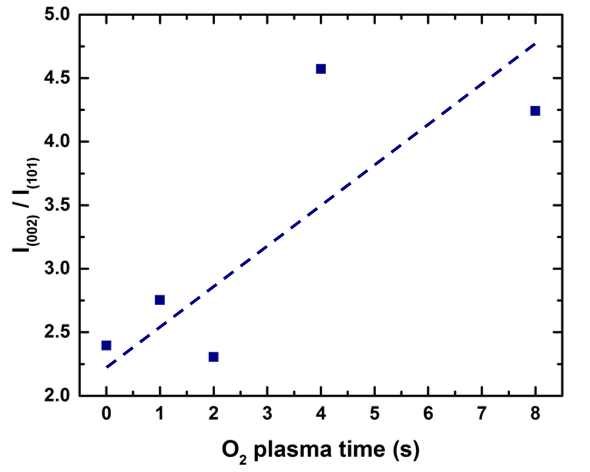


**Figure S1** Diffraction Intensity ratio of the ZnO (0 0 2) peak to (1 0 1) peak as a function of O2 plasma times with fixed thermal cycle (*m* = 1).

The optical band gap (*E_g_*) is determined by applying a Tauc’s plot, $\alpha h\upsilon=A\left( h\nu-E_{g} \right)^{1/2}$, where A is a constant, α is the absorption coefficient, and hν is the incident photon energy. The *E_g_* value can be obtained by extrapolating the linear portion to the photon energy axis in that figure. The Tauc’s plots ZnO films grown from different O_2_ plasma time with fixed thermal cycle *m* and different thermal cycle m with fixed O_2_ plasma time are as shown in Figure S1c and S1f, respectively. Similar optical band gaps at *ca.* 3.22 eV are observed for all ZnO films.


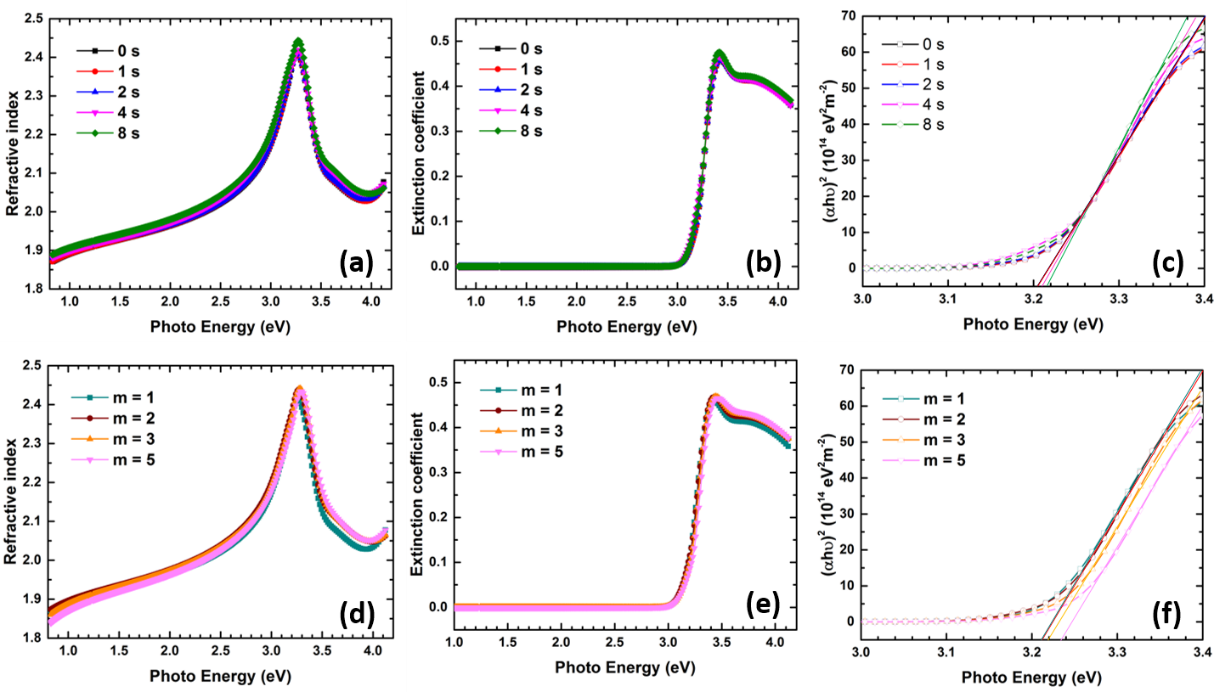


**Figure S2** (a, d) The fitted refractive index n, (b, e) extinction coefficient k and (c, f) plot of (αhν)^2^ as a function of photo energy of ZnO films grown from different O_2_ plasma times with fixed thermal cycle (*m* = 1) and different thermal cycles with fixed O_2_ plasma time (t_3_ = 1 s) by the supercycled ALD process.


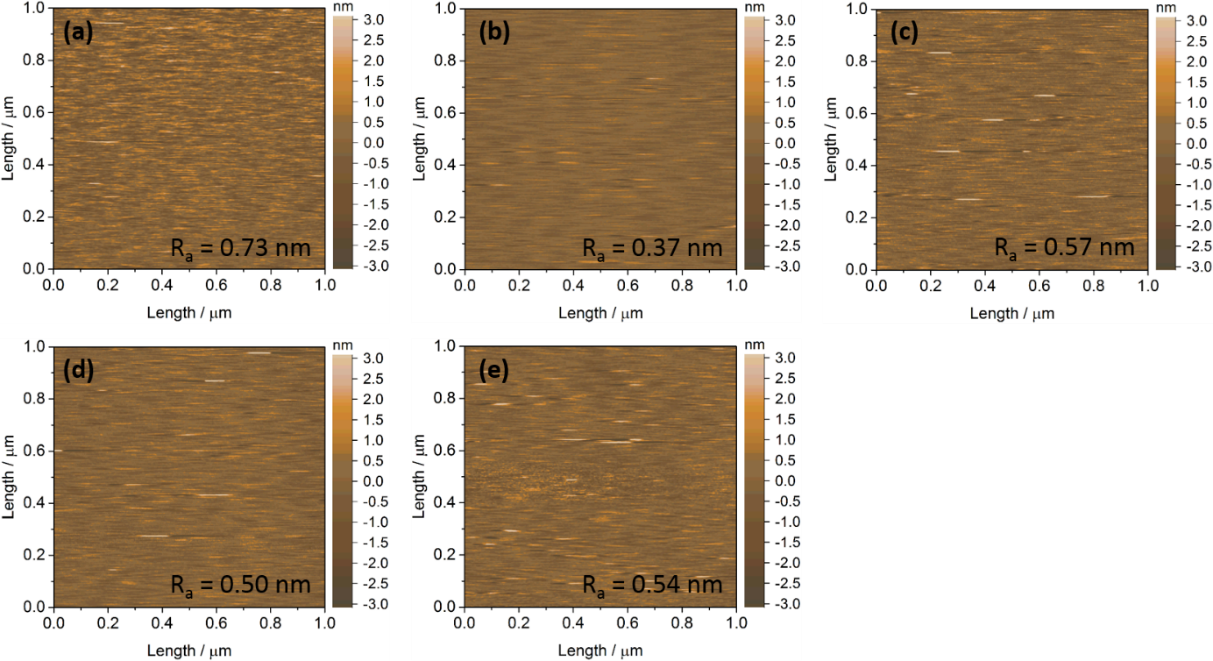


**Figure S3** AFM images of the supercycled ALD grown ZnO films with O_2_ plasma times of (a) 0 s, (b) 1 s, (c) 2 s, (d) 4 s and (e) 8 s and fixed thermal cycle (*m* = 1).

The energy level diagram in the KPFM measurement is shown in Figure 3S. Before the sample and tip are brought into connection, the vacuum levels are aligned while Fermi energy levels are different between the sample and tip (Figure S3a). Upon the electrical connection, the Fermi levels will align through a current flow to enable an equilibrium state of the system (Figure S3b). This induces surface charging over the sample and tip surfaces which leads to the built-up of the contact potential difference V_CPD_ as well as the formation of an electrostatic force between them. In the KPFM measurement, this electrostatic force is compensated by applying an external bias with the same value of V_CPD_ between the sample and tip (Figure S3c). The work function difference can therefore be obtained by the external bias value.


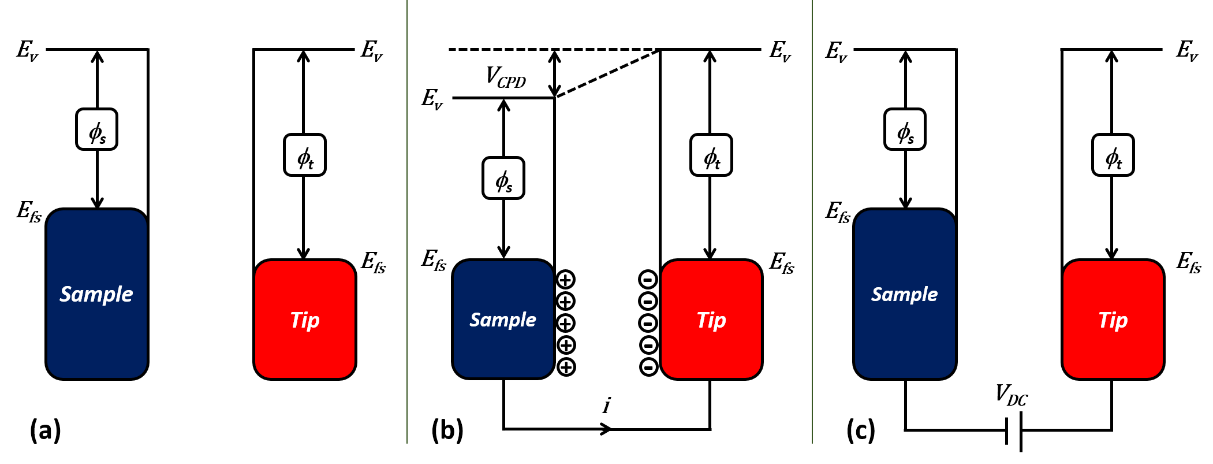


**Figure S4** Electronic energy levels of the tip-sample system for three different cases. (a) tip and sample are not electrically connected; (b) tip and sample are electrically connected with Fermi energy levels lined-up; (c) an external bias equal to the contact potential difference V_CPD_ is applied to the tip.


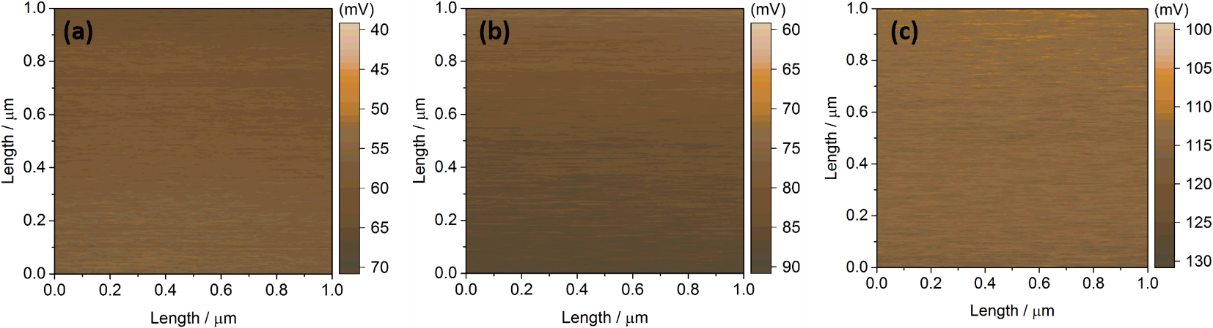


**Figure S5** Two dimensional contact potential difference V_CPD_ images of the surface potential measurements of the supercycled ALD grown ZnO films with thermal cycles varying from (a) 2, (b) 3 (c) 5 at fixed O_2_ plasma time (t_3_ = 1 s).


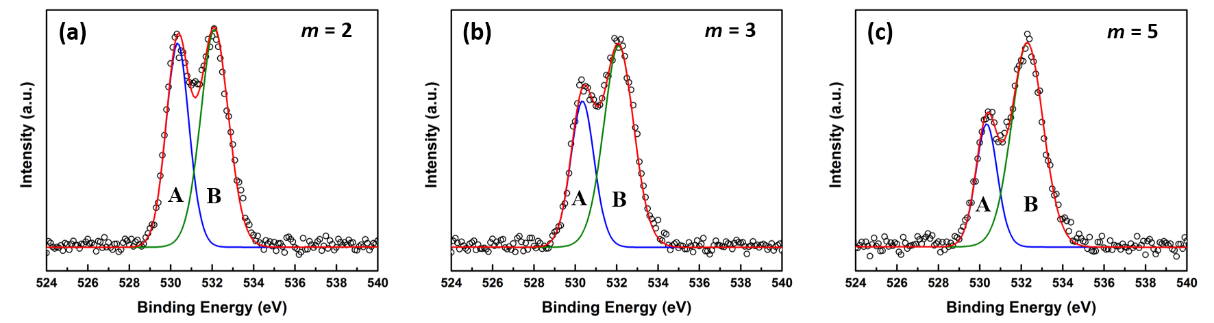


**Figure S6** XPS spectra and their Gaussian fittings of the O 1s region of the supercycled ALD grown ZnO films with thermal cycles varying from (a) 2, (b) 3 (c) 5 at fixed O_2_ plasma time (t_3_ = 1 s).
